# Epidemiological Investigation of Gastrointestinal Parasites of Dromedary Camels in Administrative Zone Three of Afar Region, Ethiopia

**DOI:** 10.1155/2022/8433997

**Published:** 2022-10-25

**Authors:** Juhar Tesfaye Bekele, Weldegebrial G. Aregawi, Fekadu Gutema Wegi, Abel Sorsa Geletu, Woldegebreil Tesfamariam

**Affiliations:** ^1^Ethiopian Institute of Agricultural Research, Werer Agricultural Research Center, Werer, Ethiopia; ^2^Ethiopian Institute of Agricultural Research, Mehoni Agricultural Research Center, Mehoni, Ethiopia; ^3^Ethiopian Institute of Agricultural Research, Holeta Agricultural Research Center, Holeta, Ethiopia; ^4^Ethiopian Institute of Agricultural Research, Wolkite Agricultural Research Center, Wolkite, Ethiopia; ^5^Ethiopian Institute of Agricultural Research, Addis Ababa, Ethiopia

## Abstract

Gastrointestinal parasites are the major threats to camel production and productivity losses in pastoral communities of Ethiopia. A cross-sectional study was conducted starting from September 2017 to April 2018 in Administrative Zone three of the Afar Region, Ethiopia. The objectives of the study were to determine the prevalence and associated risk factors for gastrointestinal parasites in camels. Fecal samples were collected aseptically from the rectum, and floatation and sedimentation techniques were used to identify the parasite in the laboratory. Out of 450 fecal samples collected from camels, 76% (71.8–79.7) of them were harboring at least one parasite in their gastrointestinal tract. The majority of infections were mixed parasitic infections. Nematodes, trematodes, protozoa, and cestodes were encountered in descending order of their prevalence. *Strongyle*, *Trichostrongylus*, and *Haemonchus* eggs were the most frequently encountered parasite eggs. The occurrence of parasite eggs was statistically significantly associated with the age of the camels and their origin (*p* value <0.05). Older camels in the Amibara district were the most likely affected groups (OR = 2.34 (1.01–5.44)). However, the sex of the camels was not associated with the prevalence of gastrointestinal parasites. Generally, the present study indicated a high prevalence of parasites which were economically important in the study area. So, awareness about the magnitude and control options should be given to the camel owners in the study area. Infected animals should be treated with effective anthelmintics like albendazole and ivermectin. Routine and scheduled deworming and good husbandry practices should be implemented. Further study on seasonal occurrences and species identification of the parasites should be studied.

## 1. Introduction

Camel is a well-known ancient mammal in the history of human civilization. It is a member of the Mammalia order, the Artiodactyla suborder, and the Camelidae family [1]. It was domesticated around 4000 years ago for transportation, meat, clothing, and milk. The meat is of excellent quality, especially in areas where other livestock animals are struggling to thrive [2]. Camel milk is comparable to cattle milk, and camels deliver milk for a longer period of time than any other domesticated animals. Animal serves as source of income and mode of transport before the invention of motorized transport and establishment of other sources of economy in areas where it was adapted [3]. Camels are bred for meat in East Africa (Kenya, Ethiopia, Sudan, and Somalia). Camels are the best-adapted species to the difficult circumstances of Ethiopia's arid/semi-arid rangelands, where pastoralism is the primary form of life and movement is an intrinsic strategy for maximizing the geographically and temporally scattered pasture and water resources [4]. Afar Region is the second most populous region and produces the most camels in Ethiopia, behind the Somali Region. Camels are primarily managed in the Afar Region for milk production, packing, and meat [5]. However, the production of camels is constrained by camel diseases in addition to other factors such as food and water shortage, less attention given, inadequate or absence of market, and inadequate veterinary services. Diseases are one of the major factors to cull camels in pastoral communities [6].

Internal and external parasites are the major problems of livestock production [7]. Camel is known to tolerate a lot of parasitic infections of economic importance among many animals with minimal economic losses. But it is also known to be infested with various helminth parasites which can cause diarrhea and other clinical signs and lead to decrease in productivity of the camels. Some of these helminth parasites also have zoonotic implication for those who work closely with camels [8]. Helminth parasites are commonly encountered from camels. *Strongyles*, *Strongyloides,* and *Trichostrongyles* are the most common nematode parasites in camels of different pastoral regions [9–11]. These parasites are also common to parasites of other livestock species which might be due to grazing on common pastures. Among gastrointestinal protozoan parasites, *Eimeria* spp. is a major problem in camel. Infection with these species might be contributing to enteric syndromes affecting camels. Coccidiosis may be seen in camel calves with symptoms like diarrhea and dysentery. There are also sign of dehydration, rough hair coat, and anemia [12]. In addition to nematodes and protozoan parasites, camels were affected by other parasites such as trematodes, cestodes, and blood parasites [11, 13, 14].

In Ethiopia, prevalence of camel gastrointestinal parasites was estimated to be high [10, 15]. The epidemiology of gastrointestinal nematode infections is influenced by climatic factors (particularly rainfall and temperature), management systems used for the animals, host factors, and parasite factors. Rainfall or moisture is the most important factor which influences the survival, development, dissemination, and availability of free living stages of helminths [11, 16].

These gastrointestinal parasites may assume much more significant role in camel husbandry because parasites not only reduce the productivity and performance of camels but also predispose them to other infectious diseases. Pastoralists in Zone three of the Afar Region have a habit of moving to highlands in search of feed for their animals during the dry seasons and due to the fear of flooding during the rainy season. As a result, it favors transmission and entrance of infections to the area [6]. Only few studies were conducted in different areas of Ethiopia but information on the magnitude and factors of gastrointestinal parasitic disease occurrence and control is still insufficient in the Afar Region particularly in Administrative Zone three. Hence, the present study was conducted to determine the prevalence and associated factors of gastrointestinal parasites of camels in selected districts in Administrative Zone three of the Afar Region.

## 2. Materials and Methods

### 2.1. Study Areas

The Afar Region is one of the regions in Ethiopia, which is divided in to five administrative zones. It is the home for 5.49 million goats, 2.75 million sheep, 1.34 million cattle, 0.77 million camels. Gabi Rasu (Administrative Zone 3) is one of the five zones of the Afar Region. This zone is bordered on the south by the Oromia Region, on the southwest by the Amhara Region, on the west by the Administrative Zone 5, on the north by Administrative Zone 1, and on the east by the Somali Region. Gabi Rasu consists of eight woredas and one town administration, including Gewane, Amibara, Galaqu, Awash Fentale (Hawaash Fantiqale), Hanruka, Dullacha, Argobba, and Awash Fentale (Hawash Subah). Amibara, Awash Fentale, and Gewane districts which are located 9°40′N 40°20′E, 9°06′N 40°02′E, and 10°9′59″N 40°38′43″E, respectively, are the three selected sites for the present study. This zone is known for its high livestock population and ranks the second next to Administrative Zone 1. Recent estimations of livestock population indicated that the zone has 1,242,335 goats, 773,499 sheep, 434,045 cattle, and 258,962 camels [5]. Camel production is one of the most important and preferred choices of pastoral communities. Camel diseases are one of the threats to their production [6].

### 2.2. Study Design and Period

A cross-sectional study design was conducted from September 2017 to April 2018 to determine the prevalence and risk factors associated with gastrointestinal parasites infecting camels in the study area.

### 2.3. Study Animals

Camels (*Camelus dromedaries*) of all age, sex, and body condition were considered as a sampling unit without discrimination. However, camels which were dewormed within a month before the study were not included in the study. Age of the animals was determined by dentition formula. Body condition scoring of the animals was performed by looking at the visible bones and muscles. Accordingly, a total of 450 camels, 118 from Awash Fentale, 130 from Gewane, and 202 from Amibara districts, were sampled.

### 2.4. Sample Size Determination

The total number of camels required for this study was calculated based on the formula given by Thrusfield [17]. Since there is no previous information in the study area, 50% expected prevalence was taken with 5% desired level of precision and 95% of confidence interval.

(1) $$\begin{array}{c} N=\frac{\left(1.96^{2}*Pexp\left(1-Pexp\right)\right)}{D\wedge 2}, \end{array}$$

where *N* = sample size; Pexp = expected prevalence; and *d* = desired absolute precision; *N* = 1.96 *∗* 1.96 × 0.5 (1–0.50)/0.05 = *N* = 384.

However, to increase the precision, additional 66 samples were taken, and a total of 450 camels were sampled.

### 2.5. Sampling and Data Collection Techniques

The study was conducted in Zone 3 of the Afar Region (Gabi Rasu). The sampling technique used was multi-stage random sampling. Accordingly, three districts were selected (Amibara, Awash Fentale, and Gewane) purposively based on their potential of camel population, accessibility of road for transport, and close proximity to Werer Agricultural Research Center (WARC). To select the representative kebeles (the smallest administrative unit in Ethiopia), discussion was made with key informants and camel owners to obtain information about the seasonal migrations of their camels. Two kebeles from each district were selected again based on the accessibility and potential of camel herds. Then, camel herds were selected from these representative kebeles randomly. Then, from each selected farm/herd, sampling animals were selected randomly and considered for the study. A total of 450 camels, proportionally from each herd, were selected and sampled.

Fecal samples were collected directly from the rectum in a separate container transported to WARC using icebox and examined for the presence of eggs using standard parasitological techniques (centrifugal sedimentation and simple floatation). For floatation, about 3 g of feces was taken from each sample and grinded with pestle and mortar within 24 hours of collection and mixed with tap water, then the mixture was sieved to test tubes and covered with cover slip on the top for five minutes, and finally the cover slip was removed gently and examined with 40x magnification. Similar procedures were followed for the sedimentation technique, but the solvent is salt solution and centrifuged for five minutes with 1500 rpm. For sedimentation procedure, the supernatant was discarded and methylene blue was dropped to stain the eggs. Then, the sediment was examined for the presence of parasitic eggs [18].

### 2.6. Data Analysis

The collected data were entered and coded on Microsoft Excel 2016 spreadsheet, and analysis was done using STATA, version 14. Proportions were used to determine the magnitude of gastrointestinal parasites. Results were described in tables and explained briefly. Chi square and binary logistic regression procedures were used to show the presence and absence and the magnitude of association of risk factors with the prevalence of gastrointestinal parasites. Odds ratios were calculated to show the degree of difference on prevalence of parasites between different groups of a factor. For all the analyses, confidence level and *p* value were declared as 95% and 0.05, respectively.

## 3. Results

Among 450 camels examined with fecal sedimentation and floatation, 342 camels were harboring one or more gastrointestinal parasite. Therefore, the prevalence was 76% (71.8–79.7) SE: 0.02 (Table 1). 63.74% of infections were mixed, whereas 36.26% of infected camels were harboring single parasite in their GIT (Table 2). Majority of the parasites encountered were nematodes (84.8), followed by trematodes (6.14), protozoa (4.1%), and cestodes (3.8%). The proportion of each parasite egg among the positive camels showed the presence of *Strongyle* egg (56.1%), *Trichostrongylus* (17.5%), *Ascaris* (7.6%), *Haemonchus* (16.3%), *Toxocara* (4.1%), *Eimeria* (4.1%), *Moniezia* (3.8%), *Strongyloides* (5%), *Dictyocaulu* (2.1%), *Ostertagia* (0.6%), *Oesophagostomum* (3.2%), *Chabertia* (0.3%), *Marshalagia* (0.3%), *Paramphistomum* (1.2%), and *Fasciola* (4.9%). The overall prevalence of parasites is statistically significantly associated with age and district of camels (*p* value <0.05), but the sex of camels was not associated with the prevalence of gastrointestinal parasites (Tables 3 and 4). Detailed descriptions of results are shown in the tables below.

## 4. Discussion

The prevalence of gastrointestinal parasites in camels in Administrative Zone 3 of the Afar Region is 76% in the present study. Consistently, other authors [9, 19] reported that 73.8% and 75.2% of camels harbor one or more types of gastrointestinal parasites in Yabelo district and Southern Ethiopia, respectively. However, the present study indicated higher prevalence than a previous research [11] which indicated the prevalence of gastrointestinal parasites to be 30.22% in the Afar Region. The prevalence of gastrointestinal parasites in camels slaughtered at Akaki abattoir which originated from different pastoral areas of Ethiopia is 55.5% [20]. Different studies out of Ethiopia were conducted and reported a prevalence of 48.26%, 60%, 17.2%, 69.3%, 50.3%, and 60.70% which are lower than those of the present study report [13, 16, 21–23]. Occurrence of gastrointestinal parasites of camels was 80.73%, and 100% in Yabelo district, and in Addis Ababa abattoir, and camels originated from Borena and Metahara, respectively [15]. In Nigeria, 92.4% prevalence of gastrointestinal parasites of camels was reported. These studies reported relatively higher prevalences of parasites compared to the present study [24, 25]. These variations on the occurrence and magnitude of parasites might be associated with husbandry practices of camel owners, availability of veterinary services, laboratory techniques used, and environmental factors (presence and absence of favorable environment).

Nematodes, trematodes, protozoa, and cestodes were encountered from the study camels with prevalence of 84.7%, 6.1%, 4.1%, and 3.8%, respectively. In line with the present study, a researcher [11] reported the highest prevalence of nematodes in camels in the Afar Region. Furthermore, the present study reported that *Strongyle* egg is the most frequently encountered parasite egg followed by *Trichostrongylus* and *Haemonchus*. Other studies indicated similar reports of *Strongyle* egg being the highest frequently encountered egg [11, 15]. However, another study [10] reported higher prevalence of *Trichostrongylus* than *Strongyles*. These indicated that nematodes are the most prevalent gastrointestinal parasites of camels.

The present study indicated that a majority (63.74%) of the infected animals were harboring two or more parasites in their GIT (mixed infections). In contrast, a researcher [19] reported the occurrence of single and concurrent infections which were 89.15% and 10.85%, respectively. However, the present study agrees with the study in Addis Ababa abattoir which reported that all of the camels were harboring at least two parasites [15]. This might be due to the presence of different variable environmental conditions, and this signifies the presence of many types of parasitic infections in Administrative Zone 3 of the Afar Region.

Districts of the camels were statistically associated with the prevalence of parasites in the present study. Accordingly, the prevalence was 80.20% in Amibara, but it was 63.08% in Gewane district (*p* value <0.05; OR = 0.37). In line with this, the authors in [16, 23] indicated that origin of camels is significantly associated with the prevalence of GIT parasites. Environmental determinants of disease such as husbandry, healthcare services, temperature, and grazing habits are variable in different locations, and therefore the prevalence of GIT parasites could be different. In contrast, camel GIT parasite infections were not associated with the district or origin of the animal in different areas of Ethiopia [20, 26].

In the present study, the prevalence of GIT parasites is highest in older camels followed by adult and young ones. The difference is statistically significant (*p* value <0.05; OR = 2.34 and 0.59). Compared to adult camels, older camels are 2.34 more likely to have GIT parasites, whereas younger ones are 41% least likely to be infected by parasites in the study area. On the other hand, the authors in [16, 20, 21] reported that there is no association between age of the animal and prevalence of parasites. It is clear that as the age of the camels increases, the exposure to different types of parasites is more and the prevalence of parasites might be higher as well. The present result is supported by different researchers stating that age and prevalence of GIT parasites are directly related in camels [9, 11, 14, 19, 22].

The prevalence of gastrointestinal parasites is higher in females (76.99%) than males (71.76%), but the difference is not statistically significant (*p* value >0.05). Many studies reported ideas similar to the present study [11, 16, 19–21]. But a study in Nigeria [22] indicated the presence of statistically significant association between sex and prevalence of parasites.

Generally, the present study indicates the presence of highly variable parasitic populations with higher prevalence and warrants the importance of implementing appropriate prevention and control measures. Unfortunately, the shortage of equipped laboratory infrastructures limited the study to go further in identifying the parasites to the species level.

## 5. Conclusion and Recommendations

The present study indicated that the overall prevalence of gastrointestinal parasites in Administrative Zone 3 of the Afar Region is high. Nematodes, trematodes, protozoa, and cestodes were encountered in descending order of their prevalence. *Strongyle*, *Trichostrongylus*, and *Haemonchus* eggs are the most frequently encountered parasite eggs. Age and district (origin of camels) showed statistically significant association with the prevalence of parasites. Thus, the occurrence is higher in older camels than adult and young camels. However, there was no statistically significant variation between the two sex groups. Considering the origin of the study camels, higher prevalence of camel GIT parasite was observed in Amibara district than in Gewane. Generally, parasitic diseases are among the most important diseases of production in the study area. Therefore, the following points are recommended.

- Treatment of sick camels with potential anthelmintics like ivermectin and albendazole should be implemented.
- Routine deworming of camels for prevention and control of gastrointestinal parasites should be practiced.
- Awareness of camel owners about the magnitude and control of these prevalent parasites should be created.
- Further studies on seasonal occurrences and species identification of the parasites should be conducted.

## Figures and Tables

**Table 1:** Prevalence of gastrointestinal parasites in camels in Zone 3 of the Afar Region.

Test result	Number of camels	Prevalence	SE	95% confidence interval	
Positive	342	0.76 (76%)	0.02	0.718	0.797
Negative	108	0.24 (24%)			
Total	450	1.00			

**Table 2:** Proportion of infection types.

Type of parasitic infection	Number of infected animals	Proportion (%)
Mixed infections	218	63.74
Single infection	124	36.26

**Table 3:** Chi square analysis on prevalence of gastrointestinal parasites of camels based on the possible risk factors.

Possible risk factors	Category of the factors	Number of camels examined	Number of camels positive (%)	Chi^2^ value	*p* value
Age	Young	185	128 (69.19)	10.61	0.005
	Adult	198	155 (78.28)		
	Old	67	59 (88.06)		

Sex	Male	85	61 (71.76)	1.03	0.31
	Female	365	281 (76.99)		

District	Amibara	202	162 (80.20)	17.07	≤0.001
	Awash Fentale	118	98 (83.05)		
	Gewane	130	82 (63.08)		

Total		450	342 (76.0)		

**Table 4:** Logistic regression analysis on degree of association between prevalence and potential risk factors.

Risk factors	Categories of risk factors	Prevalence (%)	*p* value	Odds ratio	Odds ratio (95% confidence interval)	
					Lower	Upper
Age	Young	69.19	0.03	0.59	0.36	0.96
	Adult	78.28	Reference			
	Old	88.06	0.04	2.34	1.01	5.44

District	Amibara	80.20	Reference			
	Awash Fentale	83.05	0.61	1.17	0.62	2.20
	Gewane	63.08	≤0.001	0.37	0.22	0.62

## Data Availability

The data supporting these findings are available upon request from the corresponding author.
